# Supplementary figures and images for: Hospital volume and outcomes of surgical repair in type A acute aortic dissection: A nationwide cohort study
Source: PLoS One. 2025 Jun 10;20(6):e0325689. doi: 10.1371/journal.pone.0325689 (PMC12151423; doi:10.1371/journal.pone.0325689)

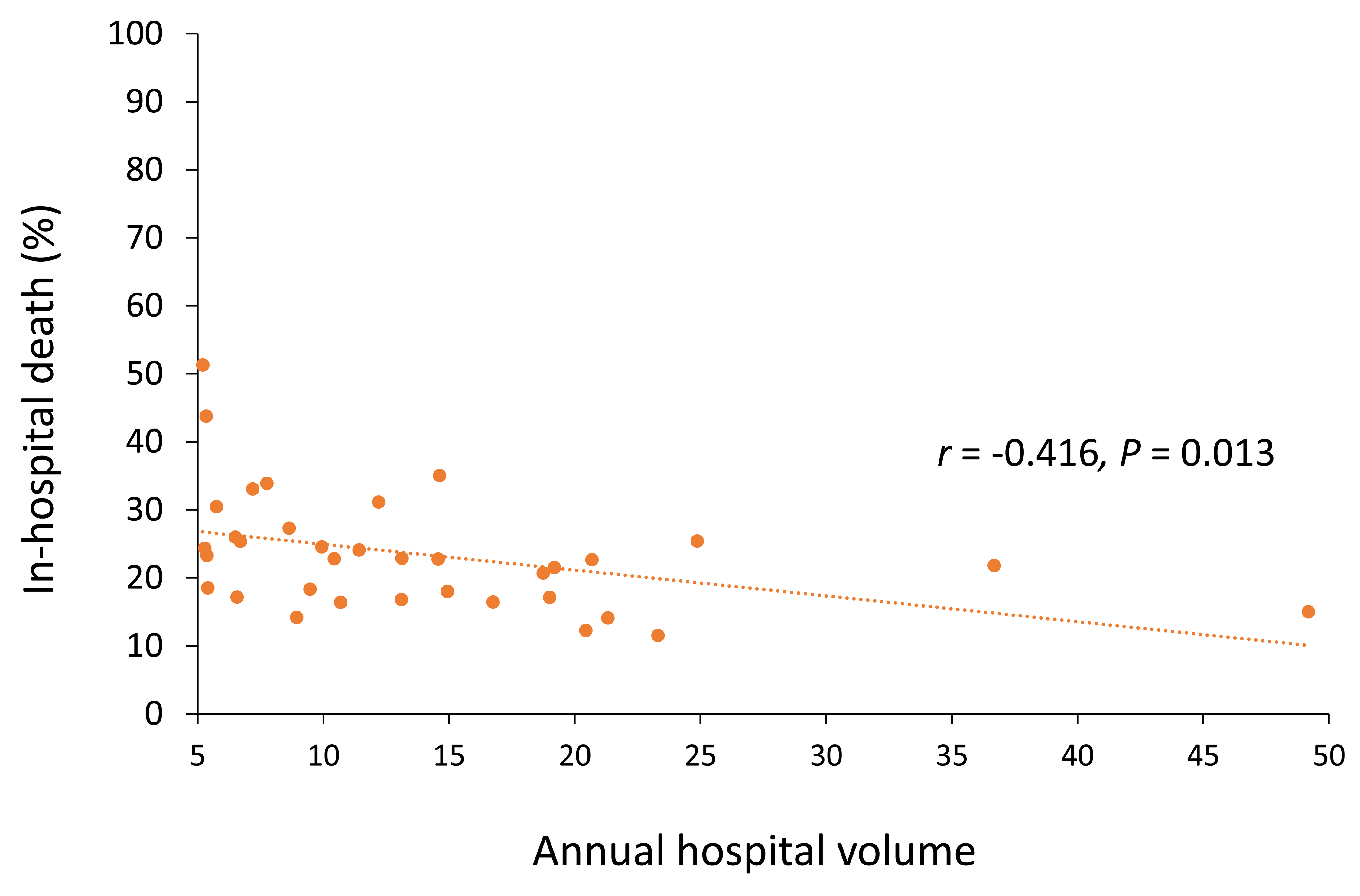

Supplement: S1 Fig — Hospitals with an annual surgical volume of five or more operations. (TIF) [file pone.0325689.s001.tif]
