# Supplementary material for: Hospital volume and outcomes of surgical repair in type A acute aortic dissection: A nationwide cohort study
Source: PLoS One. 2025 Jun 10;20(6):e0325689. doi: 10.1371/journal.pone.0325689 (PMC12151423; doi:10.1371/journal.pone.0325689)
Supplement: S1 File — Supplemental Table 1. In-hospital outcomes of the patients between those who underwent surgery at the three major centers and those treated at hospitals with a very low surgical workload (fewer than 12 operations per year). Supplemental Table 2. Late outcomes of the patients of the patients between those who underwent surgery at the three major centers and those treated at hospitals with a very low surgical workload (fewer than 12 operations per year). (DOCX) [file pone.0325689.s002.docx]

**Supplemental Table 1**. In-hospital outcomes of the patients between those who underwent surgery at the three major centers and those treated at hospitals with a very low surgical workload (fewer than 12 operations per year)

|  | Very high vs. very low volume | | Unadjusted analysis | |  | Adjusted analysis* | |
| --- | --- | --- | --- | --- | --- | --- | --- |
| Variable | The 3 major centers | <12 operations  per year | OR/*B* (95% CI) | *P* value |  | OR/*B* (95% CI) | *P* value |
| Number of patients | 1,722 | 2,628 |  |  |  |  |  |
| Primary outcome |  |  |  |  |  |  |  |
| In-hospital mortality | 347 (19.6) | 730 (27.8) | 0.63 (0.55–0.73) | <0.001 |  | 0.56 (0.48–0.66) | <0.001 |
| Secondary outcome |  |  |  |  |  |  |  |
| Cardiogenic shock and need MCS | 222 (12.5) | 317 (12.1) | 1.04 (0.87–1.25) | 0.643 |  | 0.92 (0.75–1.13) | 0.424 |
| Respiratory failure | 548 (30.9) | 433 (16.5) | 2.27 (1.97–2.62) | <0.001 |  | 2.06 (1.77–2.41) | <0.001 |
| New onset stroke | 236 (13.3) | 299 (11.4) | 1.20 (1.00–1.44) | 0.054 |  | 1.17 (0.97–1.43) | 0.104 |
| Dialysis (de novo dialysis) | 318 (18.0) | 511 (19.4) | 0.91 (0.78–1.06) | 0.213 |  | 0.85 (0.71–1.01) | 0.066 |
| Sepsis | 72 (4.1) | 150 (5.7) | 0.70 (0.53–0.93) | 0.015 |  | 0.73 (0.54–0.98) | 0.038 |
| Deep wound infection | 48 (2.7) | 59 (2.3) | 1.21 (0.82–1.78) | 0.328 |  | 1.14 (0.76–1.71) | 0.515 |
| Massive transfusion (PRBC ≥10U) | 735 (41.5) | 975 (37.1) | 1.20 (1.06–1.36) | 0.004 |  | 0.95 (0.83–1.09) | 0.462 |

Abbreviation: TAAAD, type A acute aortic dissection; Q, quartile; OR, odds ratio; B, regression coefficient; CI, confidence interval; MCS, mechanical circulatory support; PRBC, packed red blood cells;

Data were presented as mean ± standard deviation or number and percentage;

* With adjustment of all covariates listed in Table 1.

**Supplemental Table 2**. Late outcomes of the patients of the patients between those who underwent surgery at the three major centers and those treated at hospitals with a very low surgical workload (fewer than 12 operations per year)

|  | Very high vs. very low volume | | Unadjusted analysis | |  | Adjusted analysis* | |
| --- | --- | --- | --- | --- | --- | --- | --- |
| Variable | The 3 major centers | <12 operations  per year | HR (95% CI) | *P* value |  | HR (95% CI)* | *P* value |
| Number of patients | 1,722 | 2,628 |  |  |  |  |  |
| All-cause mortality | 634 (35.8) | 1,137 (43.3) | 0.73 (0.66–0.81) | <0.001 |  | 0.71 (0.65–0.79) | <0.001 |
| Mortality after discharge | 302 (17.0) | 433 (16.5) | 0.84 (0.73–0.98) | 0.023 |  | 0.87 (0.75–1.01) | 0.069 |
| Stroke | 115 (6.5) | 161 (6.1) | 0.89 (0.70–1.13) | 0.322 |  | 0.90 (0.70–1.15) | 0.391 |
| Aorta surgery |  |  |  |  |  |  |  |
| Any aorta surgery | 238 (13.4) | 225 (8.6) | 1.37 (1.14–1.65) | 0.001 |  | 1.40 (1.16–1.69) | 0.001 |
| Open repair | 64 (3.6) | 56 (2.1) | 1.46 (1.02–2.08) | 0.041 |  | 1.22 (0.84–1.77) | 0.294 |
| Stent | 192 (10.8) | 184 (7.0) | 1.32 (1.08–1.62) | 0.007 |  | 1.41 (1.14–1.74) | 0.001 |
| Re-admission due to any cause | 916 (51.7) | 1,189 (45.2) | 0.97 (0.89–1.06) | 0.469 |  | 0.97 (0.89–1.06) | 0.516 |
| New-onset dialysis | 28 (1.6) | 29 (1.1) | 1.13 (0.67–1.91) | 0.639 |  | 1.31 (0.77–2.25) | 0.320 |

Abbreviation: TAAAD, type A acute aortic dissection; Q, quartile; HR, hazard ratio; CI, confidence interval;

Data were presented as number and percentage;

* With adjustment of all covariates listed in Table 1.
