# Supplementary material for: Hospital volume and outcomes of surgical repair in type A acute aortic dissection: A nationwide cohort study
Source: PLoS One. 2025 Jun 10;20(6):e0325689. doi: 10.1371/journal.pone.0325689 (PMC12151423; doi:10.1371/journal.pone.0325689)
Supplement: Attachment — Submitted filename: 20250308 - Rebuttal Letter.docx [file pone.0325689.s004.docx]

***Reviewer #1: It is a very interesting analysis. However, do you have considered the risk indexed mortality effect during follow up period. It is not clear how you dived the quartiles, please be clear and more specific. In general all the tables should be formatted better in order to render more readable.***

***I think the phenomenon you observe is due tue pure increase of number, hence 'easy' cases prevalence which naturally bring to morality increase and experience of the surgeons, share of experience. The best way is to index based of risk and complexity of the patients and see if the adjusted mortality still follows the same phenomenon.***

Thank you very much for your valuable comments. You have highlighted a very important limitation of our study: due to the data being from the National registry, we are unable to assess disease severity or risk index mortality. However, in our analysis, all of the covariates in the regression models were adjusted to age, male sex, Marfan syndrome, hypertension, Diabetes Mellitus, atrial fibrillation, chronic kidney disease, chronic obstructive pulmonary disease, heart failure, myocardial infarction, stroke, history of cardiac surgery, year of surgery, extension of aortic surgery, partial or total aortic arch replacement, elephant trunk (conventional or frozen), aortic root replacement (Bentall operation), ascending aorta replacement only, and additional surgery (Coronary artery bypass graft, valve replacement). We have discussed this issue in the revised Limitation section (Lines 303 to 307).

Thank you for the observation. We have provided a more detailed and specific description of how we divided the quartiles in our study for analysis. We have address it in the revised ‘Study population’ subsection of the Methods section (Lines 95 to 98).

Indeed, the tables were difficult to read, we have reformatted them to landscape orientation instead of portrait. Thank you for your observation.

***Reviewer #2: The article does not add anything to what we already know. It would be appropriate to review your cut-off to be considered an aortic surgeon: 5 cases per year is not enough. I have also noticed that despite some improvement over the years the mortality rate remains at around 20%, which is still quite high. How do you explain this finding? It may be related to the fact that you have included centres with very low yearly workload and high***

***workload in the same group. It would be more appropriate to compare two separate groups: high and low workload. My understanding is that only three major centres are available. It would be worth discussing the challenges related to transfer to high volume following referral for acute and complex cases. The article would gain more value from an educational point of view if you discussed the challenging aspects and your views more extensively.***

Thank you for your valuable comments. We would like to have the space to clarify a misunderstanding. In our manuscript: line 274, first sentence. We were only trying to emphasize that when a cardiovascular surgeon performs more than five aortic surgeries/year, the mortality decreases to less than 18%. We were referring to what Norton et al(1) reported in 2022. In that document, an aortic surgeon was someone performing more than 50 aortic surgeries/year.

A 20% mortality rate represents the overall mortality rate in Taiwan (High and low volume centers). Our center, which performs more than 100 aortic surgeries/year, has a mortality of less than 10%. In the report of Biancari et al(2) in 2023, although the overall mortality was 17.7%, the hospital mortality rate varied significantly between hospitals (8.9%–30.9%). This is in general, consistent with our findings.

We agree with your opinion that only three major centers are available in our country during the study period. Therefore, we conducted a sensitivity analysis of comparing patient outcomes between those who underwent surgery at the three major centers and those treated at hospitals with a very low surgical workload (fewer than 12 operations per year). The results reinforced the primary analysis, indicating better mortality outcomes for patients who underwent surgery at major centers compared to those at low-volume hospitals. We have addressed this in the revised ‘Statistical analysis’ subsection of the Methods section (Lines 138 to 140), the ‘Sensitivity analysis’ subsection of the Results section (Lines 212 to 215), and in the newly added Supplemental Table 1 and Supplemental Table 2.

Thank you very much for the advice about the transfer issue. We have added a discussion regarding referrals for acute and complex cases(3) (Lines 280 to 285).

“However, in Taiwan, there is no regionalization policy dictating the referral of patients to high-volume hospitals instead of low-volume aortic surgery centers. Thus, despite the obvious short- and long-term benefits of referring patients to high-volume centers,(3) Taiwan’s health system does not restrict where patients can undergo surgery for acute aortic dissection. It would be worthwhile for Taiwan’s health authorities to consider pilot programs or phased approaches to regionalize the surgical treatment of acute type A aortic dissection.”

**References**

1.	Norton EL, Farhat L, Wu X, Kim KM, Fukuhara S, Patel HJ, et al. Specialization in Acute Type A Aortic Dissection Repair: The Outcomes and Challenges. Semin Thorac Cardiovasc Surg. 2023;35(3):466-75.

2.	Biancari F, Juvonen T, Fiore A, Perrotti A, Herve A, Touma J, et al. Current Outcome after Surgery for Type A Aortic Dissection. Ann Surg. 2023;278(4):e885-e92.

3.	Goldstone AB, Chiu P, Baiocchi M, Lingala B, Lee J, Rigdon J, et al. Interfacility Transfer of Medicare Beneficiaries With Acute Type A Aortic Dissection and Regionalization of Care in the United States. Circulation. 2019;140(15):1239-50.

***Reviewer #3: The Authors present an interesting research based on a large National administrative database.***

***1) unfortunately, due to inherent limitations of using administrative data, some clinical details are missing, which would be important for stratification. E.g. clinical status at presentation (shock, neurology, etc..). I understand that it's not feasible to gather such data due to the methodology of the study, which in change offers extensive follow-up information. However, this should be mentioned in the discussion section.***

***2) Centers are grouped by annual volume. Given the low numbers (10-20 surgeries/year), I suppose this is only referring to TAAAD surgery, not overall cardiac surgery activity. I think this should be clarified in the Methods section.***

***1) unfortunately, due to inherent limitations of using administrative data, some clinical details are missing, which would be important for stratification. E.g. clinical status at presentation (shock, neurology, etc..). I understand that it's not feasible to gather such data due to the methodology of the study, which in change offers extensive follow-up information. However, this should be mentioned in the discussion section.***

***2) Centers are grouped by annual volume. Given the low numbers (10-20 surgeries/year), I suppose this is only referring to TAAAD surgery, not overall cardiac surgery activity. I think this should be clarified in the Methods section.***

1) Thank you very much for the review of our manuscript. We mentioned this fact as part of our limitations. However, we have expanded the sentence to make it clearer (Lines 303 to 307):

“First, due to inherent limitations of using the Taiwan National Health Insurance (NHI) Research Database (NHIRD), we did not had access to information regarding clinical status at presentation (shock, neurology status, etc..). Disease severity was not available for study, and we did not have access to the preoperative CT scans in our study, thus, limiting stratification in our study.”

2) Thank you for your observation. We have modified the manuscript to make it clearer and avoid misunderstandings (Lines 31 to 33 within the Abstract, lines 94 to 96, and lines 148 to 149)

***Reviewer #4: An interesting paper of Iván Alejandro De León Ayala and associates. The study is devoted to the relevant problem of the relationships between the hospital-volume and surgical outcomes of type A acute aortic dissection management. The authors showed that the operative volume inversely correlates to in-hospital mortality and postoperative complications. The volume-outcome effect extends after discharge and reflects better longterm survival. Hospital referral to high-volume centers should be considered in patients needing complex open repair for type A acute aortic syndrome. Surely, it can't be said that the authors have discovered a radical new thing. In recent years, a number of publications have noted the importance of accumulating local experience in clinics that provide care for acute aortic syndrome and the need to refer patients to such centers. These indications are present in both recently published guidelines and supplementary documents, and it is advisable to specifically note this in the text. At the same time, the work under review is important both as further evidence of the need for local aortic teams and as a study that increases the body of knowledge about predictors of complications of surgery for acute type A aortic dissection.***

***The authors analyzed a huge cohort of 8,059 patients in 77 hospitals who underwent first open repair for type A acute aortic dissection from 01/01/2005 to 33 31/12/2020 in Taiwan. It is important to pay attention, to indicate possible causes (e.g., DeBakey type 2 dissection of the ascending aorta), and to supplement the discussion section with the fact that not all cases of ascending aortic prosthesis were combined with intervention on the aortic arch, at least to the extent of hemiarch reconstruction, although this approach has been recommended for quite a long time. The results showed that more annual volume was significantly correlated to a lower average in-hospital mortality of the hospital (r = -0.32), but the correlation coefficient is weak.***

***I would also recommend double-checking the text and removing annoying typos, such as “unpreparable aortic root” (lines 261-262). Regarding the reference list, I would recommend the authors remove reference #2. The paper entitled “The International Registry of Acute Aortic***

***Dissection (IRAD): new insights into an old disease”, published in 2000, looks at least strange in 2025. Otherwise, the list of references is represented by contemporary and relevant works.***

***Generally, the paper is technically sound, and the methods are appropriate and properly conducted. The claims are fully supported by the experimental data. The statistical analysis of the data is sound. The claims are appropriately discussed in the context of previous literature. In general, the manuscript is clearly written. There are no special ethical concerns from the use of human subjects. I have no other comments on the work, and I recommend it for publication after minor revision.***

***Thank you for submitting your study to PLOS One and good luck with the paper.***

Thank you very much for the appraisal of our manuscript. The typo has been corrected, and the reference #2 has been changed to a more recent publication. We appreciate the detailed feedback.

Biancari F, Juvonen T, Fiore A, Perrotti A, Herve A, Touma J, et al. Current Outcome after Surgery for Type A Aortic Dissection. Ann Surg. 2023;278(4):e885-e92.
