# Supplementary material for: Hospital volume and outcomes of surgical repair in type A acute aortic dissection: A nationwide cohort study
Source: PLoS One. 2025 Jun 10;20(6):e0325689. doi: 10.1371/journal.pone.0325689 (PMC12151423; doi:10.1371/journal.pone.0325689)
Supplement: Attachment — Submitted filename: 20250422 Rebuttal Letter.docx [file pone.0325689.s005.docx]

Response to Reviewers

Reviewer #1: Dear authors congratulations for topic.

1. Massive transfusion is defined as > 10 U, define also the single unit of transfused blood.

Thank you for the observation. A single unit equals to 450-500cc of blood. We have added this definition in line 106.

2. Are there any frozen elephant trunk cases?

As shown in Table 1, there were 1,269 (15.7%) cases of frozen elephant trunk (Few were conventional elephant trunk).

3. Quartile's division cutoff values must be also in methods section.

Thank you very much for observation. We have added the quartile's division cutoff values into the revised ‘Study population’ subsection of the Methods section. (Lines 97-98)

4. In Figure 1 C could be helpful to have linear regression line.

Thank you very much for pointing this out. We have added the linear regression line to revised Figure 1C.

5. From figure 2 is visible that hospitals with less then 5 cases year have highest mortality, visually over 50%, so I suggest to create a version of the same plot excluding hospitals with less then 5 cases, or at least to do separate plots.

Thank you for the suggestion. We have created a separate plot excluding hospitals with less than 5 cases per year. We have addressed this in the newly added Supplemental Figure and the revised ‘Sensitivity analysis’ subsection of the Results section. (Lines 213-215)

6. In figure 3 there is flipped phenomenon of cumulative mortality, it seems third quartile is doing better than the fourth one, why do you think?

This is an interesting observation. One plausible explanation is that within the highest-volume centers (4^th^ quartile), some centers handle a high volume of aortic dissections specifically but are not necessarily high-volume centers for general open-heart surgery. Consequently, their overall surgical experience and infrastructure may differ, potentially impacting outcomes negatively compared to centers proficient in both high-volume open-heart surgery and acute type A dissections. This variability could artificially elevate cumulative mortality within the 4^th^ quartile, making outcomes appear worse than the 3^rd^ quartile. Supporting this interpretation, **Figure 3B**, which excludes the two Highest-Volume Centers, shows improved cumulative mortality in the 4^th^ quartile group, indicating that these specific high-volume but less comprehensive centers may skew the overall outcomes negatively. We have described this in the revised ‘Sensitivity analysis’ subsection of the Results section. (Lines 223-229)

7. In figure 4D there is an increasing rend after around 27 cases, how you explain it?

In Figure 4D, an increasing trend after approximately 27 cases can be explained by the practice patterns of high-volume centers. Such institutions typically have more rigorous postoperative surveillance protocols, including frequent imaging studies (e.g., CT scans, echocardiograms). Consequently, they are more likely to detect indications for early re-intervention. Additionally, high-volume centers possess greater surgical experience and expertise, which makes them more proactive in scheduling and performing reoperations compared to lower-volume centers.

Reviewer #2: (No Response)

Response: thank you very much for review.

Reviewer #3: The Authors have addressed all the issues raised by the Reviewers, improving the paper throughout, therefore the article is now acceptable in my opinion.

Response: thank you very much for review.
