# Supplementary material for: Hospital volume and outcomes of surgical repair in type A acute aortic dissection: A nationwide cohort study
Source: PLoS One. 2025 Jun 10;20(6):e0325689. doi: 10.1371/journal.pone.0325689 (PMC12151423; doi:10.1371/journal.pone.0325689)
Supplement: Attachment — Submitted filename: 20250510 Rebuttal Letter.docx [file pone.0325689.s006.docx]

Response to Reviewers

Reviewer #1: Please do share your data according to PLOS data policy requirements. The patients characteristics should be in methods section. It seems that third quartile is doing better then fourth one in immediate results (table 2). Please do add a discussion on this topic. What is a meaning of table 5 excluding second and third most volume centers?

1. Response: Thank you very much for your thoughtful review. We would like to respectfully note that the issues raised have already been addressed in our previous revisions. Specifically, the points mentioned were incorporated based on earlier reviewer comments, and corresponding changes have been made in the manuscript.
